# Syphilitic abdominal aortic aneurysm – a rare case report

**DOI:** 10.1186/1471-2334-14-S3-P48

**Published:** 2014-05-27

**Authors:** R Manipriya, V Sudha

**Affiliations:** 1 Institute of Venereology, Rajiv Gandhi Government General Hospital, Madras Medical College, Chennai, India

## Background

The incidence of cardiovascular involvement in late untreated syphilis is about 10%. Syphilitic aortic aneurysm is rare since the era of antibiotics.

## Case report

A 50 year old male patient presented with abdominal pain radiating to the back for the past 6 months. He had giddiness, paroxysmal nocturnal dyspnoea. General and cardiovascular system examination showed no evidence of aortic regurgitation. Expansile epigastric pulsation with thrill was felt. Bruit was heard. No lesion or scar on genital examination.

The routine parameters were normal. VDRLwas reactive in 512 dilutions, TPHA positive, ELISA for HIV 1 and HIV 2 was negative. CT angiogram showed fusiform aneurysmal dilatation of descending aorta of length 11.6 cm with anterior wall irregularity from D11 to L2 vertebra. Coeliac and superior mesenteric arteries were seen arising from the aneurysm. Aortic arch and ascending aorta were normal. The patient was advised bed rest and treated with injection procaine penicillin1.2 million units deep i.m. after test dose for twenty days under cover of steroids. Follow up showed decreasing VDRL titres. Abdominal aortic aneurysm accounts for less than <5% of all syphilitic aneurysms. It may be misdiagnosed as atherosclerotic aneurysm. Hence serological testing is very helpful in patients diagnosed with aneurysms. CT angiogram helps to define the size and anatomy of aneurysm.

## Conclusion

This case is being reported for its rarity in the post antibiotic era and to emphasize the importance of early diagnosis and treatment to prevent complications.

